# Finding the Optimal Conditioning Regimen for Relapsed/Refractory Lymphoma Patients Undergoing Autologous Hematopoietic Cell Transplantation: A Retrospective Comparison of BEAM and High-Dose ICE

**DOI:** 10.4274/tjh.2014.0214

**Published:** 2016-08-19

**Authors:** Onur Esbah, Emre Tekgündüz, Itır Şirinoğlu Demiriz, Sinem Civriz Bozdağ, Ali Kaya, Ayşegül Tetik, Ömür Kayıkçı, Gamze Durgun, Şerife Kocubaba, Fevzi Altuntaş

**Affiliations:** 1 Ankara Oncology Hospital, Clinic of Medical Oncology, Ankara, Turkey; 2 Ankara Oncology Hospital, Hematology and Stem Cell Transplantation Unit, Ankara, Turkey

**Keywords:** Relapsed/refractory lymphoma, Hematopoietic stem cell transplantation, Conditioning regimen

## Abstract

**Objective:**

High-dose chemotherapy followed by autologous hematopoietic stem cell transplantation (AHCT) is a well-defined treatment modality for relapsed/refractory non-Hodgkin’s lymphoma (NHL) and Hodgkin’s lymphoma (HL). Although there are several options in terms of conditioning regimens before AHCT, no one treatment is accepted as a standard of care. This study aimed to compare different conditioning regimens for the treatment of NHL and HL.

**Materials and Methods:**

Medical records of 62 patients who had undergone AHCT following BEAM (BCNU, etoposide, cytarabine, and melphalan) and high-dose ICE (hICE; ifosfamide, carboplatin, and etoposide) conditioning regimens were analyzed retrospectively and compared in terms of efficacy and adverse effects.

**Results:**

The study included a total of 29 and 33 patients diagnosed with relapsed/refractory NHL and HL, respectively. Patients received BEAM (n=37) or hICE (n=25) regimens for conditioning. One-year overall survival was 73±6% in all patients. One-year overall survival was 71±8% and 74±9% in the BEAM and hICE groups, respectively (p=0.86). The incidences of nausea/vomiting (grade ≥2) (84% vs. 44.7%; p=0.04) and mucositis (grade ≥2) (13% vs. 3%; p=0.002) were higher in the hICE group compared to the BEAM group. In addition, we witnessed significantly more hepatotoxicity of grade ≥2 (40% vs. 2.7%; p<0.005) and nephrotoxicity of grade ≥2 (48% vs. 2.7%; p<0.005) among patients who received hICE. Significantly more patients (n=4; 25%) in the hICE group experienced veno-occlusive disease (VOD) compared to the BEAM arm, where no patients developed VOD (p=0.01).

**Conclusion:**

There was no difference in terms of overall survival between the BEAM and hICE groups. We observed significantly more adverse effects among patients treated with hICE. The BEAM regimen seems to be superior to hICE in terms of toxicity profile with comparable efficacy in patients with relapsed/refractory NHL and HL.

## Abstract (tr)

**Amaç:**

Otolog kök hücre nakli (OKHN) destekli yüksek doz kemoterapi relaps/refrakter non-Hodgkin lenfoma (NHL) ve Hodgkin lenfoma (HL) tedavisinde uygulanan bir yöntemdir. Hazırlama rejimleri çok çeşitli olabilse de OKHN öncesinde henüz hiçbirisi standart olarak kabul edilmemiştir.

**Gereç ve Yöntemler:**

BEAM ve yüksek doz ICE (hICE) sonrasında OKHN olan 62 hastanın tıbbi kayıtları retrospektif olarak analiz edildi ve etkinlik ile yan etki profili açısından karşılaştırıldı.

**Bulgular:**

Çalışmaya toplamda 29 relaps/refrakter NHL ve 33 HL olgusu dahil edildi. Hazırlama rejimleri BEAM (n=37) ve hICE (n=25) idi. Bir yıllık genel sağkalım (GS) %73±%6 idi. BEAM ve hICE gruplarında ise 1 yıllık GS oranı sırasıyla %71±%8 ve %74±%9 olarak bulundu (p=0,86). Bulantı/kusma (derece ≥2) insidansı (%84 vs %44,7; p=0,04) ve mukozit (derece ≥2) insidansı (%13 vs %3; p=0,002) hICE grubunda daha yüksek oranda görüldü. İlaveten, hICE alan hastalarda istatistiksel olarak derece ≥2 hepatotoksisite (%40 vs %2,7; p<0,005) ve derece ≥2 nefrotoksisite (%48 vs %2,7; p<0,005) daha fazla oranda gözlendi. hICE grubunda veno-oklüzif hastalık (VOH) sıklığı (n=4; 25%) BEAM grubu ile karşılaştırıldığında istatistiksel olarak anlamlı düzeyde daha yüksekti (p=0,01). BEAM grubunda VOH görülmedi.

**Sonuç:**

GS oranları her iki grup arasında farklı bulunmadı ancak hICE grubunda anlamlı oranda yan etki sıklığı artmıştır. Relaps/refrakter NHL ve HL hastalarında benzer etkinlik ile BEAM rejimi toksisite profili açısından hICE rejiminden üstün olarak kabul edilebilir.

## INTRODUCTION

About 50% and 20% of patients presenting with non-Hodgkin’s lymphoma (NHL) and Hodgkin’s lymphoma (HL) will not be cured after initial combination chemotherapy, respectively [1,2]. High-dose chemotherapy combined with autologous hematopoietic stem cell transplantation (AHCT) is an accepted treatment option for relapsed/refractory chemosensitive NHL/HL patients [3,4]. Predictive markers for post-AHCT outcome are chemosensitivity, number of chemotherapy lines before AHCT, disease status at the time of AHCT, relevant prognostic scores for histological subtypes of lymphoma, and time of relapse following first-line therapy (<12 months vs. >12 months) [5,6,7,8]. The best conditioning regimen before AHCT in patients with relapsed/refractory lymphoma is an undefined issue. Commonly used regimens in this scenario are BEAM (BCNU, etoposide, cytarabine, and melphalan) [8,9], BEAC (BCNU, etoposide, cytarabine, cyclophosphamide) [9], high-dose ICE (hICE; ifosfamide, carboplatin, and etoposide) [10], CMV (cyclophosphamide, melphalan, and etoposide) [11], CBV (cyclophosphamide, BCNU, and etoposide), combination regimens including total body irradiation (TBI) [12], and rituximab or I131-tositumomab combined with BEAM [13]. Few studies were reported comparing conditioning regimens in terms of toxicity and efficacy [9,13,14,15,16]. As we are unaware of any study comparing hICE and BEAM, we retrospectively analyzed our lymphoma patients who had undergone AHCT and received either hICE or BEAM regimens as conditioning.

## MATERIALS AND METHODS

### Patient Characteristics

The clinical and laboratory records of all consecutive relapsed/refractory HL/NHL patients who were treated with AHCT between 2010 and 2012 were retrospectively analyzed. We did not use any exclusion criteria. All patients gave informed consent for all aspects of AHCT and the institutional review board approved the study.

### Mobilization Strategy

We used a step-by-step mobilization strategy. Granulocyte-colony stimulating factor (G-CSF; filgrastim or lenograstim) at a dose of 10 µg/kg/day in two divided doses is our first-line mobilization protocol. A progenitor cell yield of <2×106/kg CD34+ cells was defined as mobilization failure. G-CSF alone was used in patients who received not more than two lines of chemotherapy and did not need chemotherapy for tumor control. Patients who failed mobilization with G-CSF alone, were heavily pretreated, or needed chemotherapy for debulking received G-CSF (10 µg/kg/day; filgrastim or lenograstim) plus chemotherapy for mobilization (second-line mobilization). Patients who failed two lines of mobilization received G-CSF combined with plerixafor as third-line mobilization. The details of mobilization with G-CSF plus plerixafor can be found elsewhere [17]. Patients who still failed mobilization with the aforementioned protocols received autologous bone marrow transplant.

Patients who needed second-line mobilization protocols received various chemotherapy regimens like ASHAP (doxorubicin, methylprednisolone, high-dose cytarabine, and cisplatin), R-ASHAP (rituximab-ASHAP), R-ICE (rituximab, ifosfamide, carboplatin, and etoposide), VIGEPP (vinorelbine, gemcitabine, procarbazine, and prednisone), DHAP (dexamethasone, cytarabine, and cisplatin), and cyclophosphamide (4 g/m^2^) [18,19,20,21,22].

### High-Dose Chemotherapy Regimens and Treatment Protocol

BEAM was our preferred conditioning regimen before AHCT. Currently, BCNU and intravenous melphalan are unavailable on the Turkish market. Both drugs are exported from the European Union under the supervision of the Drug and Pharmacy Agency of Turkey. We used hICE as the second choice when one or both of these aforementioned drugs were temporarily unavailable for technical reasons. The BEAM regimen included BCNU at 300 mg/m^2^ on day -7, etoposide at 200 mg/m^2^ and cytarabine at 200 mg/m^2^ on days -6 to -3, and melphalan at 140 mg/m^2^ on day -2. Patients in the hICE regimen group received ifosfamide at 2.5 g/m^2^ (total dose: 15 g/m^2^; IV infusion over 2 h), etoposide at 250 mg/m^2^ (total dose: 1.5 g/m^2^; IV infusion over 2 h), carboplatin at 250 mg/m^2^ (total dose: 1.5 g/m^2^; IV infusion over 4 h), and mesna at 3.5 g/m^2^ (total dose: 21 g/m^2^) in evenly divided daily doses on days -8 to -3.

Patients received subcutaneous G-CSF (5 µg/kg/day) from day +1 of AHCT until neutrophil engraftment (>500/mm^3^). Platelet transfusions were given if platelet counts were <10,000/mm^3^ without risk factors for bleeding. Erythrocyte suspensions were given to patients with anemia-related symptoms or hemoglobin values below 8 g/dL. All patients received levofloxacin at 400 mg/day, fluconazole at 200 mg/day, and valacyclovir at 1000 mg/day until neutrophil engraftment.

### Response and Toxicity Evaluation

Responses before and after AHCT were evaluated according to revised international working group criteria [23]. Chemosensitive disease was defined as achievement of at least partial remission (PR) following salvage chemotherapy. Chemoresistant disease was defined as inability to achieve PR or observation of progressive disease. Positron emission tomography scanning was not used. Toxicities were evaluated according to Common Terminology Criteria for Adverse Events v3.0 [24]. Follow-up examinations were carried out at day +30 after AHCT. Thereafter, surveillance examinations were done every 3 months for the first 2 years, every 6 months for the next 3 years, and then annually.

### Definition of Engraftment, Febrile Neutropenia, and Veno-Occlusive Disease

Neutrophil engraftment was defined as the first of 3 consecutive days on which the absolute neutrophil count exceeded 500/mm^3^ without G-CSF support. Platelet engraftment was defined as the first day of 7 consecutive days on which platelet count exceeded 20,000/mm^3^ without platelet transfusion [25]. We used Infectious Disease Society of America [26] and Seattle [27] criteria for defining febrile neutropenia and veno-occlusive disease, respectively.

### Management of Febrile Neutropenia

The details of our protocol can be found elsewhere [28]. Briefly, patients with febrile neutropenia who were not responding to broad-spectrum antibiotics for 72 h were evaluated for opportunistic fungal infections. Patients who had hemodynamic instability and/or two consecutive positive serum galactomannan assays (ELISA: optical density of ≥0.5) and/or thorax computerized tomography findings suggesting invasive pulmonary aspergillosis (nodules with/without halo sign, air crescent sign, and cavitation) supported by mycological cultures received antifungal treatment. Patients with mycological evidence of Aspergillus spp. were treated with voriconazole. All others received caspofungin.

### Calculation of Direct Treatment Costs of Conditioning Regimens

Direct drug costs of BEAM and hICE conditioning regimens were calculated based on an average patient with a body surface area of 1.7 m^2^ as of October 2013.

### Statistical Analysis

Descriptive statistics are presented as median and minimum-maximum. Comparisons of continuous variables between the two groups were performed using the nonparametric Mann-Whitney U test. Proportions were compared using the chi-square test. Survival analysis was calculated with Kaplan-Meier analysis. A p-value below 0.05 was considered to be statistically significant.

## RESULTS

The demographic and clinical characteristics of the study cohort are summarized in Table 1. Fifteen (40%) and 5 (20%) patients had primary refractory disease following their first-line chemotherapy in the BEAM and hICE groups, respectively (p=0.09). While 6 patients in the BEAM group received standard-dose ICE (sICE) as rescue before AHCT, no patient in the hICE arm was treated with sICE as salvage chemotherapy. Fourteen (38%) and 6 (25%) patients of the BEAM and hICE arms had refractory disease at AHCT, respectively. Twenty-six, 28, 8 patients were mobilized with G-CSF alone, G-CSF plus chemotherapy, G-CSF plus plerixafor, respectively. There were no significant differences in terms of conditioning regimens among patient groups (p=0.5 both for HL and NHL patients). The treatment arms were also similar according to age, sex, stage, previous radiotherapy, chemotherapy history, and disease status at AHCT and mobilization protocol. On the other hand, the BEAM group had significantly worse performance status compared to the hICE arm (p=0.011) (Table 1).

Mobilization success, engraftment kinetics, and side effect profiles of the conditioning regimens are given in Table 2. The BEAM and hICE treatment arms were similar in terms of infused stem cells, median days with febrile neutropenia, engraftment kinetics, and duration of hospitalization. We observed significantly more adverse effects (grade ≥2) in terms of nausea/vomiting, mucositis, hepatotoxicity, and nephrotoxicity among patients treated with hICE conditioning compared to patients who received BEAM. Significantly more patients (n=4; 25%) in the hICE group experienced veno-occlusive disease compared to the BEAM arm, where no patients developed veno-occlusive disease (p=0.01). The treatment arms were comparable according to diarrhea rate (p=0.09).

**Figure 1:**
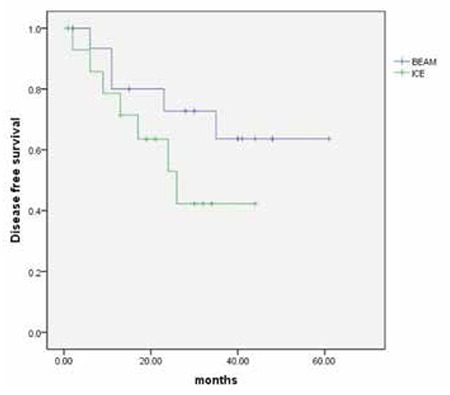
Kaplan-Meier plots of disease-free survival following autologous hematopoietic stem cell transplantation according to conditioning regimens. Three-year disease-free survival rates were 63±13% (BEAM) vs. 42±15% (hICE) (p=0.187).

**Figure 2:**
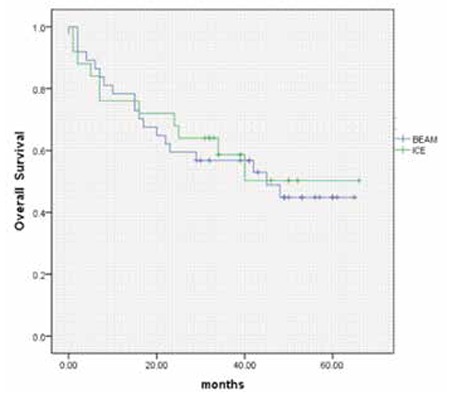
Kaplan-Meier plots of overall survival following autologous hematopoietic stem cell transplantation according to conditioning regimens. Three-year overall survival rates were 56±8% (BEAM) vs. 58±10% (hICE) (p=0.781).

Relapse rates following BEAM and hICE conditioning regimens were 13.5% (5/37) and 32% (8/25) (p=0.07). One patient of the BEAM arm died before day 30 following AHCT as a result of sepsis. Additionally, two patients (one patient in each arm) died before day 100. The reasons for mortality were sepsis/engraftment failure and Cytomegalovirus pneumonia in the patients of the BEAM and hICE groups, respectively. Transplant-related mortality for the entire cohort on day 100 was 4.8% (BEAM: 5.4%; hICE: 4%; p=0.8). Following AHCT, 5 (13.5%) and 8 (32%) patients of the BEAM and hICE arms relapsed (p=0.07). Three-year disease-free survival (DFS) and overall survival (OS) rates were 52±10% and 57±6% in the whole study cohort, respectively. There was no difference in terms of 3-year DFS rates according to conditioning regimens (BEAM: 63±13%; hICE: 42±15%; p=0.187) (Figure 1). Three-year OS was 56.8±8% and 58±10% in the BEAM and hICE groups, respectively (p=0.781) (Table 2, Figure 2).

Direct treatment costs of hICE and BEAM regimens were found to be 1721 and 582 euro, respectively.

## DISCUSSION

Although many different conditioning regimens for relapsed/refractory HL and NHL have been proposed, none of them can be considered as a standard of care [8,9,10,11,12]. Different types of hICE conditioning regimens were described according to large ranges of cumulative dosages and administrations of drugs [10,29]. To our knowledge, there is no direct comparison in the literature of BEAM and hICE chemotherapy regimens in patients who have undergone AHCT for relapsed/refractory lymphoma.

In the current study, we observed statistically significant differences in terms of toxicity favoring the BEAM regimen compared to hICE. It was not surprising that nausea and vomiting were more frequent in the hICE arm, as ifosfamide and carboplatin have high emetogenic potential. Mucositis is an important toxicity of BCNU and etoposide [30,31]. According to several studies, BCNU-related mucositis rates were higher when the BCNU dose was increased from 450 to 600 mg/m^2^ [30]. The BCNU dose in the BEAM conditioning arm was 300 mg/m^2^ in our study. The total doses of etoposide were 1500 mg/m^2^ and 800 mg/m^2^ in the hICE and BEAM arms, respectively. The higher etoposide dose may be responsible for the higher mucositis rate observed in the hICE arm. Nephrotoxicity was significantly higher in the hICE group (p<0.005). Nephrotoxicity was seen only in one patient in the BEAM group. This is not an unexpected finding because carboplatin and ifosfamide are well-known nephrotoxic agents [32]. Hepatotoxicity was also more frequent in the hICE group than the BEAM group (p<0.005). Patients on hICE experienced significantly more veno-occlusive disease compared to the BEAM arm. This may have occurred as a result of the higher total dose of etoposide in hICE compared to BEAM [31]. In addition, ifosfamide may create an extra burden on the liver, resulting in high rates of hepatotoxicity [33]. As no patient in the hICE arm received sICE as a salvage before AHCT, we think that the observed toxicity cannot be attributed to previous exposure to the same drugs at standard doses.

In Turkey, direct treatment costs of ICE and BEAM regimens in a patient with an average body surface area of 1.7 m^2^ are 1721 and 582 euro, respectively. Indirect costs of chemotherapy like hospitalization, treatment of infections, or adverse effects are not included here. The BEAM regimen was more advantageous in terms of cost.

Head-to-head comparisons of different conditioning regimens in relapsed/refractory lymphoma patients before AHCT are scarce. Jo et al. observed superior OS and event-free survival at 2 years in patients on BEAM compared to BEAC regimens (62.4% vs. 32.1% and 62.4% vs. 28.6%, respectively). However, diarrhea and mucositis were more frequent in patients of the BEAM arm [9]. In their single-center analysis, Jantunen et al. reported similar efficacy of BEAM and BEAC conditioning regimens in terms of OS and progression-free survival in patients undergoing AHCT for NHL, but BEAM was found more toxic to the gastrointestinal system [16]. In recent years the BEAM regimen was also compared with the CEB (carboplatin, etoposide, and bleomycin) regimen with better OS in favor of BEAM [14], but other studies reported conflicting results [15]. Salar et al. reported Spanish GEL/TAMO registry data including 395 consecutively autografted diffuse large B-cell lymphoma (DLBCL) patients. The main message of that study was that chemotherapy-only conditioning regimens (BEAM, BEAC, or CBV) significantly improved 8-year OS compared to TBI + cyclophosphamide [15]. Recently, the rituximab-BEAM (R-BEAM) conditioning regimen was compared to the I131-tositumomab-BEAM (B-BEAM) in a phase III randomized study in relapsed, chemosensitive DLBCL patients. Two-year progression-free survival and OS rates were comparable, but B-BEAM was found to be more toxic in terms of mucositis [13]. Although the observation period of our study cohort is limited, 3-year OS rates were similar in the BEAM and hICE arms (56±8% vs. 58±10%; p=0.781). There was a trend for lower relapse rates following BEAM compared to hICE (13.5% vs. 32%; p=0.07). There were more patients with primary refractory disease (40% vs. 20%) and refractory disease at AHCT (38% vs. 24%) in the BEAM arm compared to patients receiving hICE conditioning. The aforementioned points underline the strong antitumor effect of the BEAM regimen compared to hICE. Taking the cost and safety advantages of BEAM over hICE in addition to similar short-term DFS and OS into account, it seems reasonable to suggest that BEAM seems to be a better option than hICE for conditioning in relapsed/refractory lymphoma patients undergoing AHCT.

Our study has several limitations that make it difficult to draw firm conclusions, such as the limited number of patients, retrospective design, and heterogeneous lymphoma subtypes of the cohort. As we included patients with HL and various pathologic subgroups of NHL (DLBCL, follicular lymphoma, mantle cell lymphoma, peripheral T cell lymphoma, and anaplastic large cell lymphoma), generalization of our findings may not be appropriate for specific patient populations with lymphoma. We also had a very limited number of patients with each subtype of lymphoma, making disease-specific statistical evaluation of hICE and BEAM conditioning regimens impossible. Although the BEAM treatment arm included more patients with poor performance status, the toxicity profile of BEAM was lower compared to ICE. This point again emphasizes that BEAM is a safe and effective conditioning regimen even for patients with poor performance.

In conclusion, the current retrospective study showed that BEAM seems to be a better option compared to hICE as a conditioning regimen in relapsed/refractory lymphoma patients before AHCT with similar efficacy but low toxicity. Although there was no difference in 3-year DFS and OS, the nausea/vomiting, mucositis, nephrotoxicity, and hepatotoxicity rates were significantly higher in the hICE group compared to the BEAM group. Prospective studies with homogeneous patient populations and incorporating novel agents in the therapeutic armamentarium will be very informative in the search for the optimal conditioning regimen in specific lymphoma subtypes in the future.

## Ethics

Ethics Committee Approval: Ethical Committee approval has been not taken because it is retrospective research; Informed Consent: It was taken.

## Figures and Tables

**Table 1:**
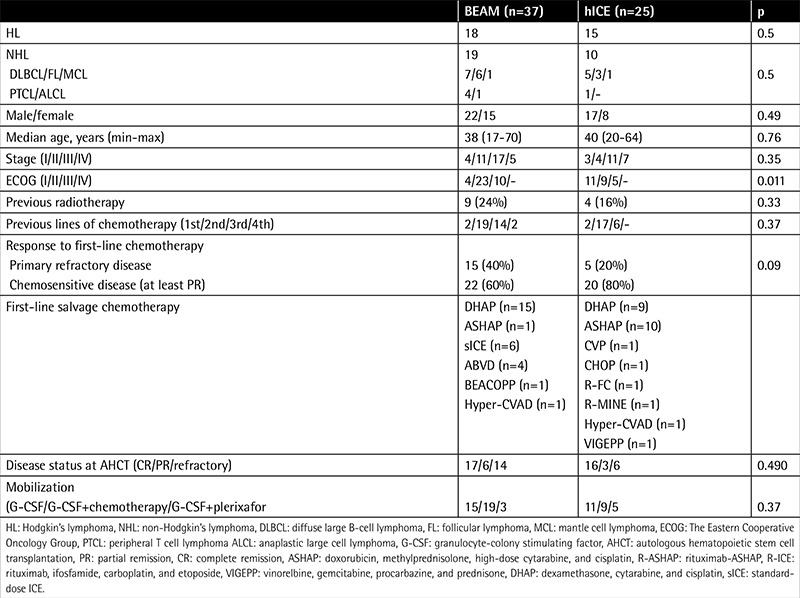
Demographic and clinical characteristics of patient cohorts.

**Table 2:**
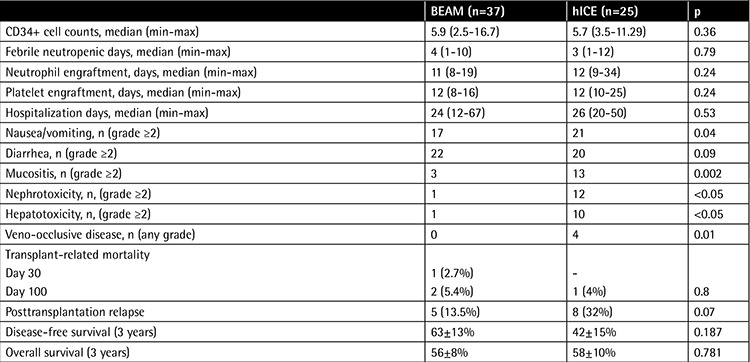
Mobilization yield, engraftment kinetics, efficacy, and toxicity profiles of conditioning regimens.
